# 

**Published:** 1925

**Authors:** 


*The following publications have been received since the publication of the List in May,* 1925.

*October,* 1925.

| | | |
|---|---|---|
| American Laryngological Association (1) | .. | 1 volume. |
| American Ophthalmological Society (2) | .. | 1 volume. |
| Mr. H. S. Bunny (3) .. .. .. | .. | 1 volume. |
| Mr. E. W. Hey Groves (4) .. | .. | 2 volumes. |
| Smithsonian Institution (5) .. .. | .. | 2 volumes. |
| University of Adelaide (6) .. .. | .. | 1 volume. |

Unbound periodicals have been received from Dr. Carey Coombs, Mr. Hey Groves, and Dr. J. Odery Symes.

### THE ONE HUNDRED AND THIRTEENTH LIST OF BOOKS.

The figures in round brackets refer to the figures after the names of the donors. The books to which no such figures are attached have either been bought from the Library Fund or received through the *Journal.*

| | | |
|---|---|---|
| **Allbutt, Sir T. C.** .. | *Arteriosclerosis* .. .. .. .. .. .. .. .. | 1925 |
| *Annals of the Pickett-Thomson Research Laboratory,* Vol. I., Part 2 | | 1925 |
| **Ashby & Earp** .. | *The Coming of Baby* .. .. .. .. .. .. .. | 1925 |
| **Beattie & Dickson** | *Beattie & Dickson's Pathology* .. 3rd Ed. | 1925 |
| **Beck, C.** .. .. .. | *The Crippled Hand and Arm* .. .. .. | 1925 |
| **Bennett, T. I.** .. | *The Stomach and Alimentary Canal in Health and Disease* .. .. .. .. .. .. .. .. | 1925 |
| *British Journal of Surgery, April and July* .. .. .. .. .. | | 1925 |
| **Campbell, T. D.** .. | *Dentition and Palate of the Australian Aboriginal* .. .. .. .. .. .. .. (6) | 1925 |
| **Cheyne, Sir W. W.** | *Lister and his Achievement* .. .. .. .. .. | 1925 |
| **Cope, Z.** .. .. .. | *Early Diagnosis of the Acute Abdomen* 3rd Ed. | 1925 |
| **Dodds & Dickens** .. | *Chemical and Physical Properties of the Internal Secretions* .. .. .. .. .. .. | 1925 |
| **Fifield, L. R.** .. | *Minor Surgery* .. .. .. .. .. .. .. .. | 1925 |
| **Flexner, A.** .. .. | *Medical Education* .. .. .. .. .. .. .. | 1925 |
| **Gibson, C. S.** .. | *Chemistry of Dental Materials* .. .. .. | 1922 |
| **Gray, A.** .. .. | *Synopsis of Gynæcology* .. .. .. .. .. .. | 1925 |
| **Groves, E. W. Hey** | *Synopsis of Surgery* .. .. .. 7th Ed. | 1925 |
| **Gullan, M. A.** .. | *Theory and Practice of Nursing* .. 2nd Ed. | 1925 |
| *Guy's Hospital Reports,* Vol. 75, No. 3, July .. .. .. | | 1925 |
| **Hill & Campbell** .. | *Health and Environment* .. .. .. .. .. | 1925 |
| **Humphris, F. H.** .. | *Artificial Sunlight and its Therapeutic Uses* 2nd Ed. | 1925 |
| **Hutchison, R.** .. | *Lectures on Dyspepsia* .. .. .. .. .. .. | 1925 |
| **Hutchison and Sherren** [Editors]. | *Index of Treatment* .. 9th Ed. | 1925 |
| **Lawrence, R. D.** .. | *The Diabetic Life* .. .. .. .. .. .. .. | 1925 |
| **Mackenzie, Sir James** | *Diseases of the Heart* .. .. .. 4th Ed. | 1925 |
| **Malgaigne, J. F.** .. | *Œuvres D'Ambroise Paré,* 3 vols .. .. | 1840 |
| **Manson Bahr, P. H.** | *Manson's Tropical Diseases* .. .. 8th Ed. | 1925 |
| **Marshall & Piney** | *Textbook of Surgical Pathology* .. .. .. | 1925 |
| **Martindale & Westcott.** | *The Extra Pharmacopeia,* Vol. II., 18th Ed. | 1925 |
| **Neuberger, Max** .. | *History of Medicine,* Vol. II., part I. .. .. | 1925 |
| **Myers, J. A.** .. | *Vital Capacity of the Lungs* .. .. .. .. .. | 1925 |
| **Ranson, S. W.** .. | *Anatomy of the Nervous System* .. .. .. | 1923 |
| **Rea, R. Lindsay** .. | *The Treatment of Interstitial Keratitis* .. .. | 1925 |
| **Runting, E. G. V.** | *Practical Chiropody* .. .. .. .. .. .. .. | 1925 |
| **Tidy, H. L.** .. .. | *Synopsis of Medicine* .. .. .. 4th Ed. | 1925 |
| **Turner, G. Grey** .. | *Some Encouragements in Cancer Surgery* .. | 1925 |
| **Weeks, C. C.** .. | *Alcohol in Medical Practice* .. .. .. | 1925 |

### TRANSACTIONS, REPORTS, JOURNALS, ETC.

Annual Report of the Smithsonian Institution, 1923 .. .. (5) 1925
Collected Papers of the Mayo Clinic, XVI., 1924 .. .. .. .. 1925
Obstetrical Transactions, Vol. XXVII., 1885 .. .. .. .. (3) 1886
Sixième Congrès de la Société Internationale de Chirurgie, July, 1923, 2 vols .. .. .. .. .. .. .. .. .. .. (4) 1925
Surgical Clinics of North America, August, 1925 .. .. .. .. 1925
Transactions of the American Laryngological Association, 46th Meeting, 1924 .. .. .. .. .. .. .. .. .. (1) 1924
Transactions of the American Ophthalmological Society, XXII., 1924 .. .. .. .. .. .. .. .. .. .. .. .. (2) 1924

### PUBLICATIONS RECEIVED.

From E. ARNOLD & Co., London:

*Health and Environment.* By LEONARD HILL, M.B., F.R.S., and ARGYLL CAMPBELL, M.D., D.Sc. 1925. Price 12s. 6d. net.

*Textbook of Surgical Pathology.* By C. JENNINGS MARSHALL, M.D., M.S. (Lond.), F.R.C.S. (Eng.), and ALFRED PINEY, M.D., Ch.B. (Birm.), M.R.C.P., M.R.C.S. 1925. Price 21s. net.

*A Synopsis of Gynæcology.* By ARTHUR GRAY, F.R.C.S., M.R.C.P. 1925. Price 10s. 6d. net.

*Lectures on Dyspepsia.* By ROBERT HUTCHISON, M.D., F.R.C.P. 1925. Price 5s. net.

From BAILLIERE, TINDALL & Co., London:

*Annals of the Pickett-Thomson Research Laboratory.* Vol. I., Part 2. June, 1925. Price 17s. net.

*Vital Capacity of the Lungs.* By J. A. MYERS, M.S., Ph.D., M.D., with Introduction by S. MARX WHITE, B.S., M.D., F.A.C.P. 1925. Price 16s. 6d. net.

From J. & A. CHURCHILL, London:

*The Diabetic Life.* By R. D. LAWRENCE, M.A., M.D. 1925. Price 7s. 6d. net.

From HEINEMANN (Medical Books) LTD.:

*The Stomach and Upper Alimentary Canal in Health and Disease.* By T. IZOD BENNETT, M.D. 1925. Price 21s. net.

From H. K. LEWIS & Co. LTD., London:

*Theory and Practice of Nursing.* By M. A. GULLAN. Second Edition. 1925. Price 9s. net.

*Alcohol in Medical Practice.* By C. C. WEEKS, M.R.C.S., L.R.C.P. 1925. Price 3s. 6d. net.

*A Preliminary Report on the Treatment of Interstitial Keratitis.* By R. LINDSAY REA, M.D., F.R.C.S. 1925. Price 2s. 6d. net.

*The Extra Pharmacopœia.* Revised by W. H. MARTINDALE, Ph.D., Ph.Ch., F.C.S., and W. W. WESTCOTT, M.B., D.P.H. Vol. II. Eighteenth Edition. 1925. Price 20s. net.

From LONGMANS, GREEN & Co., London:

*Lister and His Achievement.* By SIR WM. WATSON CHEYNE, Bt., K.C.M.G., C.B., F.R.C.S., F.R.S., etc. 1925. Price 7s. 6d. net.

From MACMILLAN & Co. LTD.:

*Arteriosclerosis.* By the late RT. HON. SIR T. CLIFFORD ALLBUTT, P.C., K.C.B., M.A. M.D. 1925. Price 5s. net.

From HUMPHREY MILFORD, Oxford University Press:

*Artificial Sunlight and its Therapeutic Uses.* By FRANCIS HOWARD HUMPHRIS, M.D. (Brux.), F.R.C.P. (Edin.), M.R.C.S. (Eng.), L.R.C.P. (Lond.), etc. Second Edition. 1925. Price 8s. 6d. net.

*History of Medicine.* By DR. MAX NEUBURGER. Translated by E. PLAYFAIR, M.B., M.R.C.P. Vol. II., Part I. 1925. Price 8s. 6d. net.

*The Chemical and Physiological Properties of the Internal Secretions.* By E. C. DODDS, Ph.D., B.Sc., M.B., B.S., and F. DICKENS, M.A., Ph.D. 1925. Price 8s. 6d. net.

*The Early Diagnosis of the Acute Abdomen.* By ZACHARY COPE, B.A., M.D., M.S. (Lond.), F.R.C.S. (Eng.). Third Edition. 1925. Price 10s. 6d. net.

*Diseases of the Heart.* By SIR JAMES MACKENZIE, F.R.S., M.D., F.R.C.P. Fourth Edition. 1925. Price 30s. net.

From THE SCIENTIFIC PRESS LTD., London:

*Practical Chiropody.* By E. G. V. RUNTING, F.I.S.Ch. 1925. Price 5s. net.

From WAKLEY & SON LTD.:

*Guy's Hospital Reports,* Vol. LXXV., No. 3, July, 1925. Price 12. 6d. net. Annual Subscription £2 2s. net.

From J. WRIGHT & SONS LTD., Bristol:

*Some Encouragements in Cancer Surgery.* By G. GREY TURNER, F.R.C.S. (Eng.). 1925. Price 7s. 6d. net.

*A Synopsis of Surgery.* By ERNEST W. HEY GROVES, M.S., M.D., B.Sc. (Lond.), F.R.C.S. (Eng.). Seventh Edition. 1925. Price 17s. 6d. net.

*The British Journal of Surgery.* Vol. XIII. No. 49. July, 1925. Price 12s. 6d. net. Yearly Subscription 42s. net.

*An Index of Treatment.* By Various Writers. Edited by ROBERT HUTCHISON, M.D., F.R.C.P., and JAMES SHERREN, C.B.E., F.R.C.S. Ninth Edition. 1925. Price 42s. net.

*A Synopsis of Medicine.* By HENRY LETHEBY TIDY, M.A., M.D., B.Ch., F.R.C.P. Fourth Edition. 1925. Price 21s. net.

### Bristol
### Medico-Chirurgical Journal.

Published Quarterly (demy 8vo)
under the auspices of the Bristol
Medico-Chirurgical Society.

---

*An excellent means of advertising*

---

The only Medical Journal published in the West of England, and has an extensive circulation not only amongst the Medical men of Bristol, but also amongst those of the adjoining Counties and South Wales, and in exchange goes to all parts of the world.

Terms for Advertisements:

Whole Page, £2 2s., Half Page, £1 5s., Quarter Page, 15s.
per issue.

PRICES FOR SPECIAL POSITIONS ON APPLICATION.

J. W. ARROWSMITH LTD.,

Advertising Agents,

11 Quay Street,

BRISTOL.

